# Adiponectin alleviates blood hypercoagulability via inhibiting endothelial cell apoptosis induced by oxidative stress in septic rats

**DOI:** 10.22038/IJBMS.2018.29389.7117

**Published:** 2018-10

**Authors:** Yun Hou, Xi-Feng Wang, Zhi-Qiang Lang, Wei Zhao, Yinchuan Jin, Hong-Qin Zhang, Lian-Shuang Zhang

**Affiliations:** 1 Department of Histology and Embryology, Binzhou Medical University, Yantai, P R China; 2 Department of Critical Care Medicine, Yu Huang Ding Hospital, Qingdao University, Yantai, P R China; 3 Department of Pathology, Yu Huang Ding Hospital, Qingdao University, Yan Tai, P R China

**Keywords:** Adiponectin, Apoptosis, Endothelial cells, Oxidative stress, Rats, Sepsis, Thrombophilia

## Abstract

**Objective(s):**

The purpose of this stu

dy was to detect the protective effects of adiponectin on coagulation dysfunction and its mechanism in sepsis of rats.

**Materials and Methods:**

The experimental samples were composed of sham group, model group that was underwent cecal ligation and puncture (CLP) and three adiponectin treatment groups that treated by adiponectin with different dose (72 μg/kg, 96 μg/kg and 120 μg/kg) after CLP. The prothrombin time (PT), activated partial thromboplastin time (APTT) was measured, respectively, the level of malondialdehyde (MDA), tissue factor (TF), activated coagulation factor VIIa and Xa, p-selectin were detected, the histology structure of vascular was observed, the expressions of Caspase 9, Caspase 3, Bax, Bcl-2 and vWF in vascular were measured.

**Results:**

The results demonstrated that adiponectin treatment lengthened PT and APTT, reduced the expression of MDA, TF, activated coagulation factor VIIa, Xa and p-selectin in plasma of septic rats. Additionally, adiponectin treatment alleviated endothelial cell apoptosis and oxidative stress, down-regulated the levels of Caspase 3, Caspase 9, Bax, Bcl-2 and vWF in vascular.

**Conclusion:**

These findings suggest that adiponectin treatment might be a promising therapeutic strategy for relieving septic endothelial cell injury and coagulation dysfunction via inhibiting endothelial cell apoptosis in septic rats.

## Introduction

Sepsis with serious infection is urgent in intensive care unit (1-3). It has been showed that the morbidity of sepsis increased annually in the past years (4). Moreover, shock induced by sepsis is still the major causes of death in US (5, 6), but it lacks effective prevention and treatment strategies. 

Microcirculatory dysfunction is known to be a critical element of sepsis and septic shock (7), which is indeed characterized by a procoagulant state, leading to disseminated intravascular coagulation (DIC) (8, 9). Sepsis patients commonly suffer from DIC and microthrombus formation (10). In addition, the occurrence of coagulation is closely related to endothelial cells which located in the innermost layer of blood vessels. Altered endothelial properties may be involved in microcirculatory failure and contributes to high mortality in sepsis (11), since the endothelium provides an interface between inflammation and coagulation (12). Moreover, oxidative stress is important for endothelial dysfunction or TF production (13). Under some pathological states like sepsis, oxidative stress accounts for endothelium injuries and the increase of TF production (14, 15).

So, in view of these mechanisms, finding an effective strategy for paitients therapy is important. In present, the uses of antibiotic and target therapy are important in the management of sepsis (16-19). However, they are limited in the clinical applications (20). Adiponectin is a cytokine released from adipocytes and play a protective role in the progress of inflammatory and oxidative stress (21, 22). It is reported that adiponectin could improve the function of vascular endothelium (23). Moreover, the previous study confirmed that the levels of microvascular inflammation in APN-/-mice were significantly reduced by supplementation of globular adiponectin (24). In conclusion, these studies indicate that adiponectin plays a protective role in sepsis. Therefore, the addition of exogenous adiponectin may be therapeutic for the sepsis treatment. However, there are few reports on whether adiponectin has endothelial protection and its mechanism in sepsis.

Hence, the current research was to investigate the effect of adiponectin on blood coagulation and its mechanism by measuring oxidative stress and endothelial cell apoptosis in septic rats.

## Materials and Methods

***Experimental design***

The operation procedure and animal use conform to the Animal Ethics Committee of Binzhou Medical University. Sepsis was induced in Wistar rats (Male, 8-week-old). The experiment consists of 5 groups, sham group (n=15), CLP model group (n=15), CLP +adiponectin (72 μg/kg) treatment group (n=15), CLP+adiponectin (96 μg/kg) treatment group (n=15) and CLP+adiponectin (120 μg/kg) treatment group (n=15). The CLP model making reference our previous experiments (25) and the selection of the adiponectin dose is based on the results of the pre-experiment. 

After 12 hr of CLP, rats in each group were euthanized with 7% chloral hydrate (5 ml/kg). The whole blood samples were sent to the hospital for testing PT and APTT using specific kits (Moshake Biotechnology Company, Wuhan, China ) according to the instructions. The remaining plasma and vascular tissue from each animal were collected and stored at -80 ^°^C for subsequent analysis.

***Detection of MDA, P-selectin, TF, coagulation factors Xa and VIIa in plasma***

The MDA concentration was measured using a MDA kit (JianCheng Biotechnology Company, Nanjing, China). The procedure reference its instructions and our previous study (26), the absorbance of each samples was read with microplate reader (Product model: DNM-9602G, China) at 532 nm. 

The plasma levels of P-selectin, TF, coagulation factors Xa and VIIa were determined using ELISA kits (YuChen Biotechnology Company, Shanghai, China). The samples of plasma were added into 96-wellplates; then, the procedure reference its instructions and our previous study (26). The absorbance at 450 nm was shown with microplate reader (Product model: Thermo Multiskan MK3, USA).

**Figure 1:**
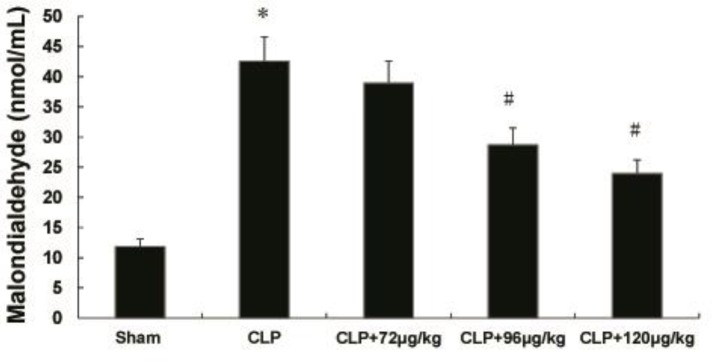
Effects of adiponectin on plasma levels of malondialdehyde in septic rat. (mean±SEM). * indicates difference compared with sham group (*P<*0.05), # indicates difference compared with CLP group (*P<*0.05)

**Figure 2:**
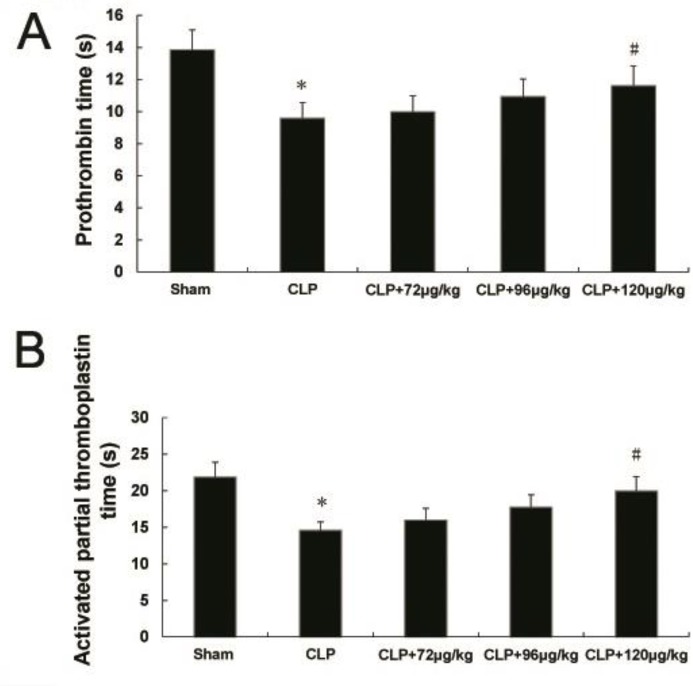
Effects of adiponectin on the prothrombin time (A) and activated partial thromboplastin time (B) in the blood. * indicates difference compared with sham group (*P<*0.05), # indicates difference compared with CLP group (*P<*0.05)

**Figure 3:**
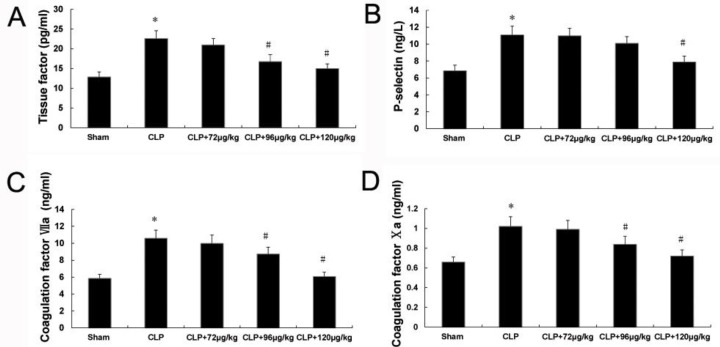
Effects of adiponectin on the coagulation factors. The levels of TF (A), p-selectin (B), coagulation factor VIIa (C) and Xa (D) in plasma. * indicates difference compared with sham group (*P<*0.05), # indicates difference compared with CLP group (*P<*0.05)

**Figure 4:**
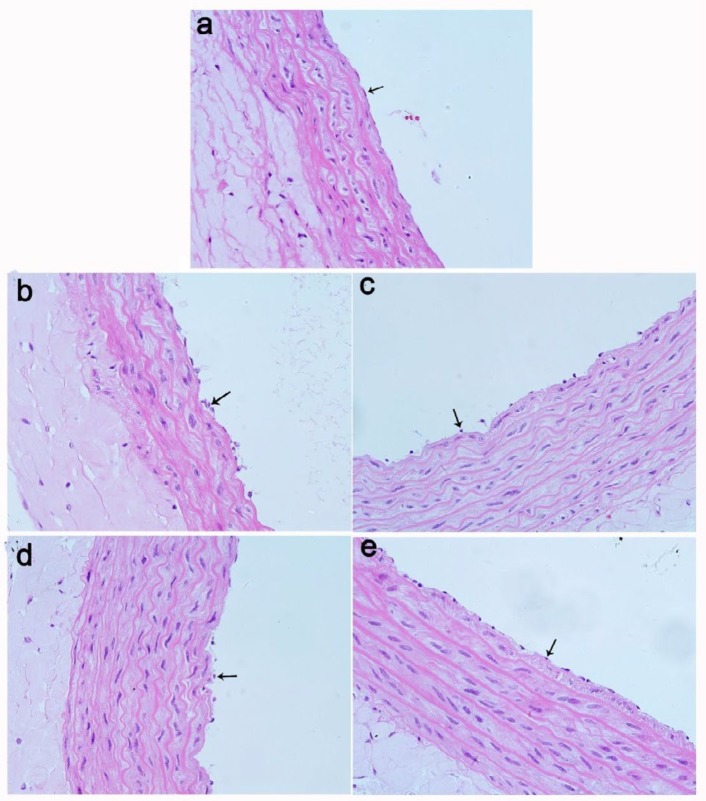
Histopathological observation of artery in rats. Sham group (a), CLP group (b), 72 μg/kg group (c), and 96 μg/kg group (d) and 120 μg/kg group (e). Black arrows denote endothelial cell. HE ×400

**Figure 5:**
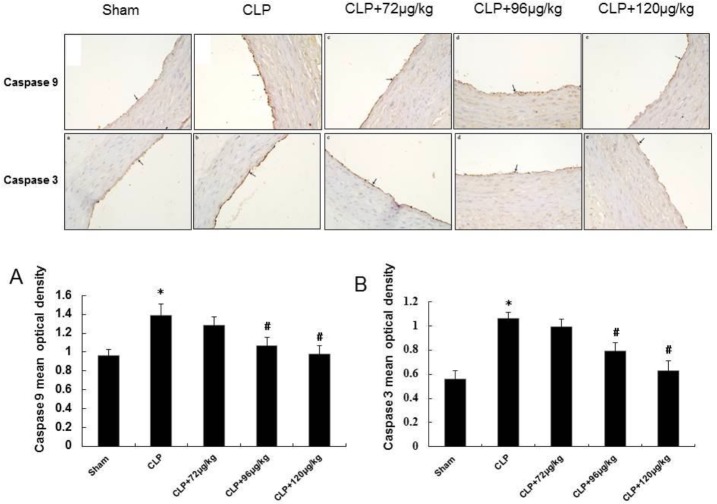
The expression of Caspase 9 and Caspase 3 in endothelium of each group. Black arrows indicates endothelial cell. Immunohistochemistry ×400. The mean optical density of Caspase 9 and Caspase 3 (Figure 5A, B), * indicates difference compared with sham group (*P<*0.05), # indicates difference compared with CLP group (*P<*0.05)

**Figure 6:**
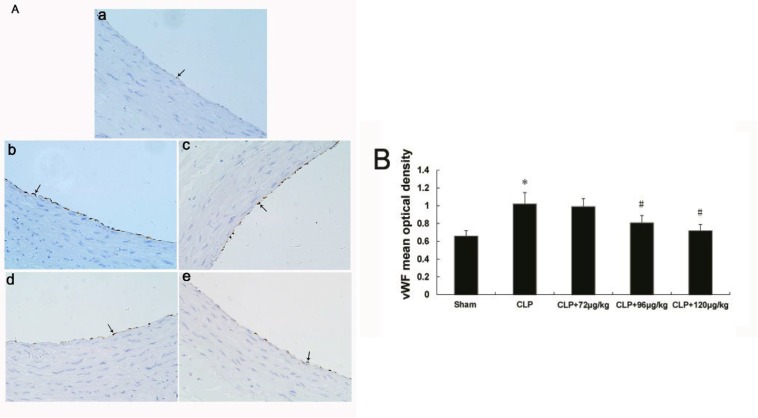
The expression of von Willebrand factor in endothelium of each group. Sham group (a), CLP group (b), 72 μg/kg group (c), 96 μg/kg group (d) and 120 μg/kg group (e). Black arrows indicates endothelial cell. Immunohistochemistry ×400. The mean optical value of vWF, (B), * indicates difference compared with sham group (*P<*0.05), # indicates difference compared with CLP group (*P<*0.05)

**Figure 7:**
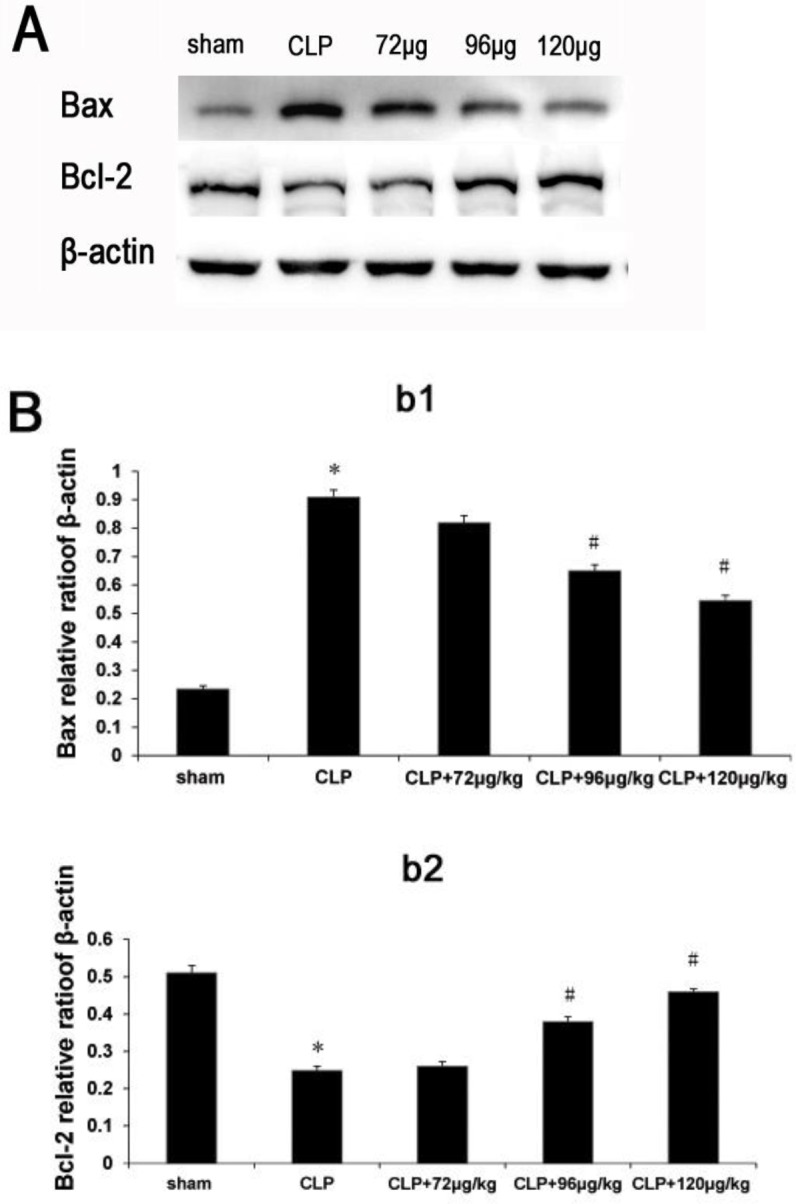
The protein levels of Bax and Bcl-2 in each group. A. Expressions of Bax and Bcl-2. B. The values of Bax and Bcl-2. * indicates difference compared with sham group (*P<*0.05), # indicates difference compared with CLP group (*P<*0.05)

**Figure 8:**
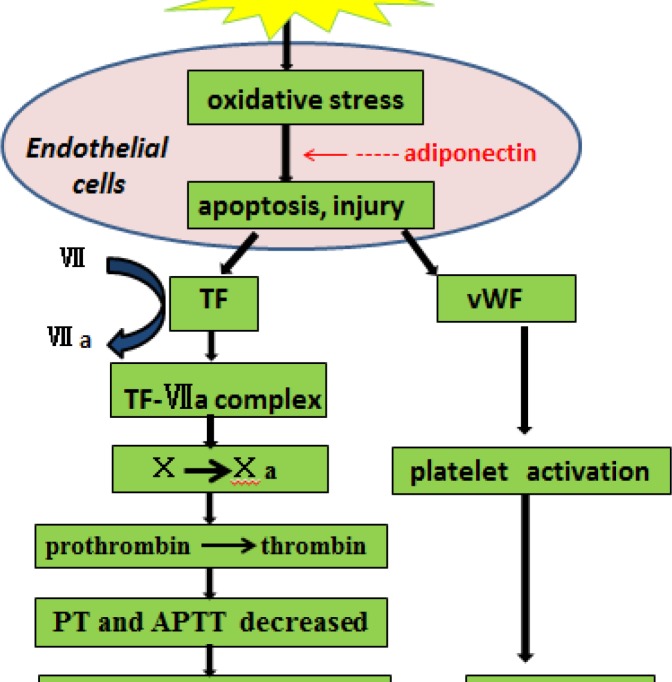
Schematic diagram for protective effect of adiponectin on coagulation dysfunction. Oxidative stress induced by inflammation resulted in the apoptosis and injury of endothelial cell which released vWF and TF into blood. Coagulation cascade reaction and hypercoagulable status of blood was induced. However, adiponectin relieves the hypercoagulable state of the blood by reducing oxidative stress. It indicated a protective effect of adiponectin on endothelial cells

***Hematoxylin and eosin (HE) staining***

The large artery of abdomen was fixed in fixation fluid and dehydrated with different concentration of alcohol, and then, the samples were embedded in paraffin and sectioned at 4 μm (Leica RM2245, Germany), stained with HE according to standard procedures, the arteries were observed under a light microscope (Olympus X71-F22PH, Japan) after staining.

***Immunohistochemistry staining for Caspase 9, Caspase 3 and vWF***

Vascular tissue were incubated in the antibodies (Caspase 9, 1:500, vWF, 1:1000 and Caspase 3, 1:1000) at 4 ^°^C overnight. And then, the sections were washed and incubated with secondary antibody. In the last, it stained with 3-3-diaminobenzidine substrate and observed under the bright field microscopy, analyzed with a computer image analyzing system (Product model: Motic Images Advanced 3.2).

***Measurement of protein expression***

Each sample of 20 μg proteins was electrophoresed and transferred to polyvinylidene fluoride membranes, the membrane was incubated with Bax (1:1000, CST, USA), Bcl-2 (1:1000, CST, USA) respectively at 4 ^°^C overnight after blocking. Then, the membrane was washed and incubated with secondary antibody for 1 hr. The protein were assessed by chemiluminescence reagent (Thermo) and analyzed with the Image Software (Bio-RadLaboratories Inc., Hercules, CA, USA).

***Statistical analysis***

The SPSS 17.0 was used to perform the statistical analysis. All data were analyzed using one-way ANOVA and followed by LSD test. Values are expressed as means ±standard.

## Results

***Changes of MDA levels in plasma***

The MDA level in CLP group was higher when compared to sham group. But decreased both in CLP+96 μg/kg and CLP+120 μg/kg adiponectin treatment groups (Figure 1).

***Changes in coagulation time of PT and APTT***

The PT and APTT in CLP group were lower than that of the sham group. But no difference was observed in CLP+72 μg/kg and CLP+ 96 μg/kg adiponectin groups compared with CLP group, these were higher in CLP+120 μg/kg adiponectin treatment group when compared to CLP group (Figure 2).

***Changes of P-selectin, TF, Xa and VIIa levels in plasma***

The therapy effects of adiponectin on TF, coagulation factors VIIa and Xa was similar. The plasma levels of these proteins in CLP group were higher compared to sham group. But all decreased both in CLP+96 μg/kg and CLP+120 μg/kg adiponectin groups compared with CLP group (Figure 3A. 3C. 3D).

Moreover, compared to the sham group, the levels of p-selectin in CLP group significantly increased, and it significantly decreased only in CLP+120 μg/kg adiponectin group compared with CLP group (Figure 3B).

***Changes in vascular tissue structure***

The artery of sham group showed regular endothelium with normal nucleus and cytoplasm (Figure 4A). But in CLP group, pyknosis of nuclear and abnormality of cytoplasm were observed in endothelial cells, and some cells exfoliated from the tunica intima (Figure 4B). The endothelial cell structure of adiponectin at 72 μg/kg (Figure 4C) and 96 μg/kg (Figure 4D) groups showed small improvement compared to CLP group. However, the damage of endothelial cells has improved some in the 120 μg/kg adiponectin treatment group (Figure 4E). Therefore, adiponectin played a protective role on endothelial cells.

***Changes in Caspase 9 and Caspase 3 expression in vascular tissue***

As shown in Figure 5, Caspase 9 and Caspase 3 immunopositive reactant distributed in cytoplasma of endothelial cells, and the expressions of Caspase 9 in CLP group significantly increased compared with sham group, but decreased both in CLP+96 μg/kg and CLP+120 μg/kg adiponectin treatment groups when compared to CLP group (Figure 5).

***Changes in vWF expression in vascular tissue***

As shown in Figure 6A a-e, endothelial cells in vascular showed vWF immunopositive, and the expressions of vWF in CLP group increased significantly compared with sham group, but decreased both in CLP+96 μg/kg and CLP+120μg/kg adiponectin groups when compared to CLP group (Figure 6B).

***Changes of Bax and Bcl-2 protein levels in vascular***

As the Figure 7A, 7Bb1 showed, the expression of Bax in CLP group increased compared with sham group, but decreased both in CLP+96 μg/kg and CLP+120 μg/kg adiponectin groups when compared with CLP group. 

As shown in Figure 7A, 7Bb2, the expression of Bcl-2 in CLP group decreased compared with sham group, but increased both in CLP+96μg/kg and CLP+120μg/kg adiponectin groups compared with CLP group.

## Discussion

Coagulation dysfunction is a notable symptom of sepsis. It was directly associated with DIC and septic shock contributing to a high mortality of critical ill patients in intensive care units (27). Endothelial injuries are also closely related to coagulation disorder of septic shock (28), numerous factors such as TF were released from damaged endothelial cells would contribute to pro-coagulant properties in sepsis (29-31). Increasing evidence has shown that TF is the principal initiator of thrombin generation (32) and inhibition of it attenuates coagulopathy and improves survival in experimental models of sepsis (33). Thus, early protection of endothelial cells could be of great interest for therapy of sepsis. 

Adiponectin is secreted from adipose tissue and endogenous adiponectin play protective effects against hypertensive vascular injury (34). Previous studies have shown that its concentration are lower in some cardiovascular disease (33), and also reduced in mice subjected to CLP (35), one recent research showed that mortality have increased in adiponectin-null mice following CLP (36). The goal of our study was to extend these findings and determine the effect of adiponectin on coagulation time which were evaluated with PT and APTT assay.

The present experimental results showed that PT and APTT were shorter in CLP group, however, adiponectin reversed it at the dose of 120 μg/kg. These data also fit with previous studies that reduced adiponectin levels in mouse increase procoagulant endothelial cell activation during sepsis (38). To prove this, the other factors related to coagulation pathway were assessed. The results indicated that in the CLP group, the levels of coagulation factors Xa, VIIa and TF significantly increased in plasma, which is in accordance with the value of PT and APTT. The present results indicate that the pathway of coagulation was activated. VIIa binds TF to form the complex which then induced coagulation cascade in blood (39). In the same time, factor X was also activated into Xa by TF-VIIa complex (40), other factors were activated by factor TF, Xa and initiate blood coagulation subsequently (41). 

In addition, vWF could be released from Weibel-Palade bodies of endothelial cells (42).The histopathological results showed that endothelial cells exfoliated from the tunic intima which may lead to the release of vWF into blood. Indeed, the expression pattern of vWF similar as TF that was gradually increased in CLP group, which accelerated the activation of platelet (43). In addition, the level of P-selectin is a marker of platelet activation. The present results indicate that P-selectin increased in CLP group, which is in accordance with the levels of vWF (44). 

Thus, CLP induces a hypercoagulable status of blood through activating the extrinsic coagulation pathway. However, the coagulation state was alleviated by adiponectin which down-regulated the secretion of clotting factors. These data show that the treatment of adiponectin has a protective effect for coagulation dysfunction in sepsis.

In order to further detect its mechanisms of adiponectin protective role for endothelial cell, apoptosis and oxidative stress mechanisms were detected. LPS is known to induce oxidative stress, enhance the formation of ROS and lipid peroxidation products such as MDA in the development of sepsis (45-46). The results demonstrated that CLP induced a high levels of MDA consistenting with the report that elevations in MDA following CLP in rats (47). MDA could mediate cell damage by controlling the expression of Bcl-2 family proteins and caspases (48). It has been investigated that Bcl-2/Bax ratio reduction induced by LPS accelerated cell apoptosis (49-52). However, adiponectin has alleviated endothelial cell apoptosis through inhibiting oxidative stress as the previous studies that it has the effect of anti-oxidative stress (53). 

Our results found that oxidative stress resulted in apoptosis and injury of endothelial cell in sepsis, and then TF was released and activated extrinsic coagulation pathway. However, the supplement of exogenous adiponectin could relieve the hypercoagulable status of blood via reducing oxidative stress induced apoptosis and injury of endothelial cells (Figure 8). Therefore, it has protective role for endothelial cells in sepsis. Finally, this study provides an experimental basis for application of adiponectin in clinical treatment of sepsis, but its uses for septic patients remain to explore.

## Conclusion

The present study investigated that adiponectin has a potential vascular protective effect in a dose-dependent manner to alleviate blood hypercoagulability via inhibiting endothelial cell apoptosis induced by oxidative stress in septic rats.
